# Challenges in Realism-Based Ontology Design: a Case Study on Creating an Ontology for Motivational Learning Theories

**Published:** 2021

**Authors:** Irshad Ally, Werner Ceusters

**Affiliations:** 1 Department of Biomedical Informatics, University at Buffalo, Buffalo, NY, USA

**Keywords:** motivational theory, learning theory, realism-based ontology, basic formal ontology

## Abstract

**Objective:**

to identify on the basis of a use case major problem types novices in realism-based ontology design face when attempting to construct an ontology intended to explain differences and commonalities between competing scientific theories.

**Methodology:**

an ontology student was tasked (1) to extract manually from a paper about five distinct motivational learning theories the scientific terms used to explain the theories, (2) to map these terms where possible to type-terms from existing realism-based ontologies or create new ones otherwise, (3) to indicate for new type-terms their immediate subsumer, and (4) to document at every step issues that were encountered.

**Results:**

where term extraction and type-term assignment were handled satisfactorily, correct classification in function of the BFO was a major challenge. Root causes identified included ambiguous and underspecified term use in the theories, the ontological status of psychological constructs, lack of high quality ontologies for the behavioral sciences and insufficient ‘deep’ understanding of some BFO entities, in part because of insufficient documentation thereof suitable for learners. The issues the student encountered were often insufficiently described for the instructor to identify the problem without analyzing the source paper itself.

**Conclusion:**

whereas behavioral scientists need to do efforts to make their theories comparable, realism-based ontologies can help them therein only when ontology developers and educators put more effort in making them more accessible without violating the principles.

## 1. Introduction

Ontological Realism [1] is a methodology that seeks to represent the entities that are denoted by the theoretical terms [2] in scientific theories. It is used the create and update the upper ontology ‘Basic Formal Ontology’ (BFO) [3]. These entities are represented through the creation of representational units (RUs) that are elucidated or defined using Aristotelian definitions of the form ‘*S* =*def. a G which Ds*’ [3, p69] and that together form a graph-theoretical structure for which an *isA* relation defined in terms of a primitive *instanceOf* relation functions as hierarchical backbone. Additional well-defined relations may provide further connections between the RUs and be used in formal axioms that represent the axioms, suppositions and hypotheses of a specific theory. Strict adherence to the realist methodology and the additional principles of the Open Biomedical Ontologies (OBO) Foundry [4] is recommended to avoid duplication of effort, to facilitate reuse and combinability of domain ontologies, and when applied correctly, comparability of data repositories that are annotated in their terms.

However, adherence to ontological realism for the representation of scientific theories is challenging, not only because it requires a thorough understanding of the entities recognized at the level of the BFO [5], but also because scientific theories rarely make assertions outside the realm of their specific domain, let alone up to the level of the BFO. Matters become even more complicated for domains where competing theories exist, and where these theories use terms which at first sight denote the same types of entities, but upon more detailed inspection do not! This is exactly the problem in behavioral science as claimed by Hastings *et al.*: ‘*In the behavioural sciences, one key challenge in adopting any theory is the plethora of competing alternative entities and the field’s lack of a principled approach to integrate across or select between them for use in interpreting a given phenomenon.*’ [6]. In this paper we highlight challenges trainees in ontology design may encounter when attempting to develop a realism-based ontology to compare motivational learning theories as discussed in *Motivation to Learn: an overview of contemporary theories* by Cook and Artino [7].

## 2. Background

Understanding motivations to learn has many potential applications, particularly in education where understanding how motivation works may contribute to developing more effective educational strategies [8]. There is no shortage in motivational learning theories, the most prominent types thereof being Expectancy-Value Theories (EVT), Attribution Theories (AT), Social-Cognitive Theories (SCT), Goal Orientation Theories (GOT) and Self-Determination Theories (SDT). Theories which fall under these categories differ in the importance they attach to various possible drivers for motivation: expectation of success and perceived value thereof, explanations learners create for their achievements or lack thereof, self-efficacy for a learner to support self-regulated learning, learners’ mindsets with respect to various sorts of goals and, finally, intrinsic interests weighted against extrinsic values which satisfy basic psychosocial needs of autonomy, competence and relatedness. However, a major problem is that ‘*the diversity of theories creates confusion because most have areas of conceptual overlap and disagreement, and many employ an idiosyncratic vocabulary using different words for the same concept and the same word for different concepts*’ [7, p998]. Although Cook and Artino were able to outline where overlap and disagreement exist, they concluded that resolving conceptual differences requires more than dictionary definitions alone and that *‘the degree to which these differences can be both theoretically and empirically reconciled remains to be seen*’ [7, p1011].

## 3. Methods

As part of a 500-level 3- credit faculty-mentored research rotation taken in parallel with another 500-level course on the principles of realism-based ontology design, the first author of this paper, a graduate student, assessed under the guidance of the second author, his instructor in the course, the feasibility to develop a ‘Motivational Learning Ontology’ (MLO) for the salient entities from the various theories described in Cook and Artino’s paper, which henceforward will be referred to as ‘the MLT paper’, ‘MLT’ standing for ‘motivational learning theories’. The goal of the MLO would be to improve comparability of these theories and to contribute to the realism-based ontologies dealing with mental functioning [9, 10]. To build such an ontology, the following activities have to be carried out: (A1) identification in the motivational learning paper of all the scientific terms used to describe the various theories, (A2) where possible, classification of the entities represented by these terms by means of the representational units already present in the BFO or in domain ontologies using the BFO as upper ontology, (A3) creation of new representational units where no suitable representational units are found in existing ontologies, including the classification thereof in terms of what is already represented and finally (A4) the formulation of axioms for what is considered invariant in each of the motivational learning theories. Further required to reach the stated goals are the development of (A5) a terminology that is anchored in the ontology and that maps the terminology used in the individual theories to the representational units of the ontology thereby thus highlighting synonymous and homonymous use of terms across, and possibly even within theories, and (A6) a collection of relational expressions that formalize the commonalities and differences between the individual theories.

In light of the educational experience being offered, within a time frame too small to carry out the activities A1 to A6 completely – an endeavor that is still ongoing – the student was specifically tasked to focus on A1, A2, A3 and A5 leaving A4 and A6 for after taking an advanced 700-level course in biomedical ontology development. He was also asked to create an inventory of the sorts of problems he encountered while performing the activities.

The student completed the task in the following manner. He began by reviewing the explanatory diagrams that were provided for each theory (figures 1 to 5 in the MLT paper) and put the terms found therein into a table thereby annotating each term with the theories in which it was found. Using the terms identified in the first table, the student then created a second table to identify the portions of reality (PORs) denoted by each term in the first table based on the descriptions provided in what in the MLT paper is called ‘Table 2‘ and which summarizes the commonalities and differences amongst the theories, thereby potentially being a good source for detecting synonyms and areas of potential conceptual conflations. This resulted in additional terms being included in the analysis. Each term was then further annotated with a type-term figuring as potential candidate for later inclusion as representational unit in the MLO. Type terms were maximally reused from existing realism-based ontologies when they were already represented there; and they were otherwise created. As this table was intended to capture synonymy and homonymy amongst terms, each type-term was further annotated with the theories with which it was associated. As a last step, the student created a third table containing for each new type-term a direct subsumer which could either be taken from an existing realism-based ontology, or a more specific type-term selected from the second table.

The tables, together with the annotations concerning the problems the student encountered, were then analyzed by the instructor, an experienced ontologist and major contributor to the BFO, having some background, but not expert, knowledge about the learning theories. The instructor’s role was to review and score and provide feedback on the student’s performance in the three (3) tasks, i.e., term extraction, assignment of type terms, and linking to potential subsumers. Importantly, the instructor did not review the paper in full with the goal (1) to determine the extent to which the student was aware of problems and pitfalls, and (2) to identify, where possible, the root causes for these issues, f.i. unclarities in the MLT paper or the theories therein described, areas where the student needs further education in the realism-based ontology development principles or where these principles themselves are not sufficiently documented in the relevant literature.

## 4. Results

Term extraction from the individual theory diagrams and comparison table produced 143 distinct terms and, coincidentally despite the occurrence of homonymy and synonymy, the same number of type-terms. In total 157 distinct term/type-term occurrences were formed of which in 39% of cases the proposed type-term was different than the term encountered in the MLT paper, typically more precise and specific than the original term. The distribution of the extracted terms over the various learning theories is depicted in Table 1. 9 terms were found to be ambiguous intra-theory, while for 4 type-terms a total of 11 synonyms was used in- and across theories. Terms marked as ambiguous and/or being used across theories are listed in Table 2 together with their occurrence in specific theories. Ambiguous use was most prevalent in GOT.

Table 3 provides tallies for the type-terms for which the student reported an issue, either when trying to determine what sort of entity was intended by a scientific term or when trying to determine for the type-term a direct subsumer, compared to the number of issues the instructor encountered while judging the adequacy of the proposed subsumers. Since 9 type-terms were taken immediately from an existing ontology, 134 out of 143 judgements had to be made. The instructor agreed with 51 of the decisions made and had an issue with 58. He wasn’t able to decide on 25 proposed subsumers on the basis of the information provided by the student, i.e. without the need to analyze the MLT paper itself. Table 3 shows for the definite and probable issues identified by the instructor also their distribution over the various theories. Since specific issues may occur in more than one theory, the sums of these distributions are larger than the number of issues. It is SDT which exhibited the most issues while GOT showed the least despite a higher degree of ambiguity in its description in the MLT paper.

## 5. Discussion

### 5.1. Barriers and pitfalls from the student’s perspective

Although a detailed analysis of the issues student and instructor encountered has not been carried out at this time, certain trends are observable. While extracting relevant terms from the various theory descriptions didn’t seem to be problematic, identifying what sorts of entities they might denote under a realist perspective, if any at all, turned out be much more cumbersome.

One recurrent theme is term ambiguity, an issue which is recognized in the behavioral sciences and which was one motivation for Cook and Artino to write their MLT paper. Ambiguity could sometimes be resolved, but more often not. Attribution theory, for example, uses the term ‘task difficulty’. Yet, the MLT paper fails to clarify whether this refers to an objective task difficulty inherent in the task itself, or if it is referring to a subjective task difficulty, as interpreted by the person performing the task. EVT recognizes ‘perceived task difficulty’ as an entity, yet it remains unclear whether it is synonymous with ‘subjective task difficulty’ and depends on whether ‘perception’ is to be interpreted in the neuroscience sense, thus being originated in sensorial input, or more general as a mental representation whether or not resulting from sensorial input. Another example in AT is the term ‘effort’ which is used variably for a ‘perceived cause’, i.e. the effort a learner believes to be required to learn something, or ‘observed behavior’, i.e. the effort a learner is actually committing in trying to assimilate new knowledge. Ontologically, these two ‘efforts’ are of very different sorts, as are subjective and objective task difficulty. It would therefore be a mistake to classify ‘perceived effort’ and ‘actualized effort’ as subtypes of ‘effort’, and ‘subjective task difficulty’ and ‘objective task difficulty’ as subtypes of ‘task difficulty’. Under a realist perspective, the former ones are cognitive representations, thus continuants, and the latter ones occurrents.

Task A2 turned out to be challenging due to the lack of existing BFO-compliant ontologies with appropriate representational units for reuse. The BioPortal contains a few ‘ontologies’ which cover part of the behavioral science domain, but these are in reality not more than vocabularies in disguise, consisting of lists of thousands of terms without any hierarchy (Psychology Ontology, Cognitive Atlas Ontology) or just one level (Behavioral Change Technique Taxonomy). While the definitions provided in these vocabularies can help to understand the domain and identify ambiguities, they provide no help for realism-based classification. A notable exception is the Emotion Ontology [10] of which in particular the appraisal subhierarchy turned out very useful, either for reuse, or as inspiration for creating type-terms following similar principles. This allowed the student to circumvent the lack of a causal theory in BFO by creating representational units that refer to cognitive representations about (assumed) causal relations, rather than dealing with these causal relations directly.

### 5.2. Student / instructor differences

Whereas the instructor didn’t identify major issues with the manual term extraction and type-term assignments proposed by the student, he disagreed with 43% of the proposed type-term subsumers and flagged another 19% thereof as questionable. For both categories as well as for the correctly proposed subsumers (38%) the student identified issues (either with respect to his understanding of the domain or of the realism-based ontology principles, or with respect to unclarities in the source document) in roughly 50% of cases (Table 3). This means that for the correct assignments, the student was able to make the correct decision despite the identified issues, but that for the doubtful and incorrect subsumer assignments, he in half of the cases was either not aware of any issue, or was sloppy in documenting his thought process. Lab notes are indeed as important in ontology design, as in wet lab experiments. Definite mistakes most often encountered include (1) misclassification as generically dependent continuant and (2) the distinction between realizable entities and qualities. Subsumer assignments assessed as doubtful by the instructor were either due to the absence of lab notes or caused by the instructor’s awareness of distinct philosophical views relevant to the problem at hand and for which in BFO no particular stance is taken. This is specifically the case for the wide variety of psychological constructs used in behavioral sciences in general [11] and learning theories in particular for which it remains to be seen whether they (or which ones) directly intend existing entities or are mere forms of speech [12].

## 6. Conclusions

Where term extraction and type-term assignment were handled satisfactorily, correct classification in function of the BFO was a major challenge for a number of reasons: ambiguity and underspecification of terms used in the theory descriptions, the ontological status of psychological constructs and their relation to the BFO, lack of high quality ontologies for the behavioral sciences and insufficient ‘deep’ understanding of some BFO entities, in part because of insufficient documentation thereof and problems for the student to understand the first-order logic axiomatizations, a topic not covered in the 500-level course. The issues the student encountered were often insufficiently described for the instructor to identify the problem without analyzing the source paper itself.

The findings reported here suffer from some limitations: (1) it involves only one student and one instructor, (2) neither student and instructor are experts in motivational learning theories, (3) the student has only 6 months part-time experience in realism-based ontology design principles but is instructed by a major contributor to the BFO and the principles of ontological realism, and (4) detailed analysis of the lab notes is still ongoing. We nevertheless dare to conclude that whereas behavioral scientists studying learning might need to do some efforts to make their theories comparable, realism-based ontologies can help them therein only when realism-based ontology developers and educators put more effort in making them more accessible without violating the principles. Anticipated next steps are therefore changes to the student learning objectives in the aforementioned 500-level biomedical ontology course, improving the documentation about BFO and ontological realism, and more extensive collaboration between ontologists and domain experts.

## Figures and Tables

**Table 1:** Scientific terms extracted from five motivational learning theories

	EVT	AT	SCT	GOT	SDT

Terms extracted	23	38	20	29	47
• Recognized homonymous intra-theory	0	1	1	5	2
• Recognized synonymous intra-theory	1	0	1	1	1

**Table 2:** Occurrence of ambiguous terms (‘Amb’) and terms used in multiple theories (UDT) across motivational learning theories

Extracted term	Amb	UDT	EVT	AT	SCT	GOT	SDT

ability	N	Y	0	1	0	1	0
achievement	N	Y	1	1	0	0	0
affect (emotion)	N	Y	0	1	1	0	0
choice	N	Y	1	1	0	0	1
engagement	N	Y	1	1	0	0	0
expectancy for success	N	Y	1	1	0	0	0
goal	N	Y	1	0	1	0	0
observable behavior	N	Y	1	1	0	0	0
performance	N	Y	1	1	1	0	0
persistence	N	Y	1	1	0	0	0
reward	N	Y	0	0	1	0	1
effort	Y	Y	1	2	0	0	0
challenge (growth)	Y	N	0	0	0	2	0
challenge (risk)	Y	N	0	0	0	2	0
cognition	Y	N	0	0	2	0	0
excessive challenge	Y	N	0	0	0	0	2
exert extra effort	Y	N	0	0	0	2	0
helplessness	Y	N	0	0	0	2	0
optimal challenge	Y	N	0	0	0	0	2
withhold effort	Y	N	0	0	0	2	0

Number of ambiguous terms in theory			0	1	1	5	2

**Table 3:** Comparison of student issues with instructor issues and distribution thereof across theories

Instructor identified issue	Student identified issue		Number of instructor issues found in theory				
	Y	N	EVT	AT	SCT	GOT	SDT

Maybe (25)	13	12	5	5	5	2	16
No (51)	24	27					
Yes (58)	29	29	9	18	5	6	24

Totals (134)	66	68	14	23	10	8	40
